# Influenced tumor microenvironment and tumor immunity by amino acids

**DOI:** 10.3389/fimmu.2023.1118448

**Published:** 2023-01-31

**Authors:** Min Yu, Shuang Zhang

**Affiliations:** ^1^ Department of Thoracic Oncology, West China Hospital, Sichuan University, Chengdu, Sichuan, China; ^2^ Department of Biotherapy, Cancer Center, West China Hospital, Sichuan University, Sichuan, Chengdu, China

**Keywords:** tumor microenvironment, amino acids, tumor immunity, T lymphocytes, cancer cells

## Abstract

It is widely accepted that tumors are a complex tissue composed of cancer cells, extracellular matrix, inflammatory cells, immune cells, and other cells. Deregulation of tumor microenvironment promotes tumor aggressiveness by sustaining cell growth, invasion, and survival from immune surveillance. The concepts that some dietary nutrients could change tumor microenvironment are extremely attractive. Many studies demonstrated that high-fat diet-induced obesity shaped metabolism to suppress anti-tumor immunity, but how amino acids changed the tumor microenvironment and impacted tumor immunity was still not totally understood. In fact, amino acid metabolism in different signaling pathways and their cross-talk shaped tumor immunity and therapy efficacy in cancer patients. Our review focused on mechanisms by which amino acid influenced tumor microenvironment, and found potential drug targets for immunotherapy in cancer.

## 1 Introduction

Deregulation of cellular metabolism is a prevalent hallmark of cancer. Normal cells primarily rely on mitochondrial oxidative phosphorylation to have energy needed for cellular engineering. However, cancer cells rely on aerobic glycolysis characterized by high glucose consumption and lactate production, which is termed as “the Warburg effect” (1, 2). Growing evidence indicated that oncogenic mutations could change the pathways in metabolism and constitutively increase the uptake of nutrients, which promote cancer cells survival and invasion (3). However, the tumor was not only a group of cancer cells, but rather a heterogeneous collection of extracellular matrix and cellular compartments such as malignant, immune, stromal, and endothelial cells. Along with nutrient consumption and metabolite production, tumor cells shaped the metabolic milieu and signaling interactions among the cellular compartments (4–6). The nutrients in tumor microenvironment had a profound effect on tumor metabolism and tumor immunity, and the nutrients in tumor tissue might differ from that in normal tissue (7).

Many dietary interventions such as chronic caloric restriction for cancer therapy were in clinical practice (8). One study found that the AMPK coupling SENP1-Sirt3 signaling promoted T cell survival, and glucose limitation was the key signal to stimulate AMPK signaling and then activated SENP1-Sirt3 axis in T cell memory development (9). Meanwhile, the ketogenic diet decreased the amount of glucose and had therapeutic effects in several types of cancers. The increased ketone bodies under ketogenic diet activated CD8^+^ T cells relying on OXPHOS. Furthermore, CD8^+^ T cells in response to ketogenic diet secreted more cytokines, such as tumor necrosis factor-α and IFN-γ (10–13). In glucose-deprived conditions, macrophages shifted towards the M2-like phenotype and showed decreased glycolysis, promoting cancer progression in various types of tumors (14).

High body fat was associated with an increased likelihood of multiple malignancies (15). Aberrant lipid metabolism was observed in many cancers, which maintained phospholipids production to cancer cell membranes and suppressed anti-tumor immunity. One study demonstrated that tumor cells and CD8^+^ T cells showed completely distinct metabolic characteristics to obesity. Tumor cells increased fat uptake under high-fat diet, whereas tumor-infiltrating CD8^+^ T cells did not increase fat uptake, and the differential adaptations led to change fatty acid profile in tumors, impairing CD8^+^ T cell infiltration and anti-tumor effects (16, 17).

Amino acids were fundamental nutrients for protein synthesis, but the mechanism amino acids supported various immune cell functions was little explored until now. In our review, we discussed the potential roles of individual amino acids in tumor immunity, which could be combined with other therapeutic agents to maximize effectiveness of tumor immunotherapy.

## 2 Overview of amino acids

While caloric restriction could enhance cancer therapy, low protein diets had also been shown to decrease cancer development (7, 18). The protein restriction might regulate IGF-1 levels and decrease overall mortality and cancer death risk in the 65 and younger population. Meanwhile it indicated that animal proteins restriction appeared to be better than plant proteins restriction, suggesting that specific amino acids content in the diet was more important than overall protein intake in tumor immunity and tumor therapy (19).

An important evolutionary step was the ability to combine a wide variety of amino acids into peptides and proteins, which performed cellular functions. Beside protein synthesis, some amino acids were considered to be involved in many other processes including proliferation signal transmission. The amino acids were classified as essential or non-essential, and some amino acids might be conditionally essential depending on cellular metabolic state and the microenvironment (20). Amino acids were essential nutrients for both tumor and immune cells. T cells heavily relied on amino acid metabolism for their maturity, differentiation, and function. Tumor cells used amino acids not only for their proliferation and invasion, but also for strategies of tumor immune evasion (21, 22).

Amino acids were transported across the plasma membrane by amino acids transporters that belonged to the solute carrier superfamily, which was composed of a large variety of members. The amino acid transporters could be classified into neutral, acidic, and basic classes. One amino acid could be transported by different transporters, meanwhile, one transporter might transport multiple substrates (20, 23). One study indicated a downregulation of acid transporter SLC6A14 and increased acid transporter SLC1A2 in endocrine therapy resistant breast cancers. The impaired amino acid metabolism enhanced specific amino acids import mediated by the SLC1A2 transporter, which provided potential targetable pathways to combat endocrine therapy resistant breast cancers (24). However, the same transporter might have different abundances in different cell types (25, 26), such as immune cells and tumor cells, which raised the challenge in biologic and translational research targeting amino acid carriers.

## 3 Amino acids in tumor microenvironment

### 3.1 Alanine

Alanine was the second-most abundant amino acids in plasma after glutamine, and was synthesized from pyruvate by alanine transaminase 1/2 (ALT1/2). Recent studies indicated that alanine was important for early T cell activation and memory T cell restimulation. However, due to limited expression of ALT1/2 needed for alanine biosynthesis, T cells depended on extracellular alanine for protein synthesis, and consumption of exogenous alanine by cancer cells might impair T cell function. Serum alanine concentrations fluctuated in mammals depending on the dietary composition and feeding schedule, and control of local alanine levels through the uptake might impact T cell activation. Meanwhile, alanine availability in tumor tissue might affect anti-tumor immunity, and interventions targeting alanine uptake might provide the therapeutic strategy for increasing T cell activation (27, 28). One study used adult zebrafish harboring BRAFV600E-driven melanoma to detect the effect of cancer on distant tissues. Melanoma consumed 15 times more glucose than other tissues measured. Excretion of glucose-derived alanine in tumors provided the source of carbon for hepatic gluconeogenesis and removed excess nitrogen from branched-chain amino acid. Pharmacological inhibition of the tumor-liver alanine cycle in zebrafish decreased tumor burden (29).

### 3.2 Arginine

Arginine was a non-essential amino acid derived from the diet or *de novo* synthesis. It had been known that some human cancers had low expression of argininosuccinate synthase 1 (ASS1). Cancer cells were unable to synthesize arginine from citrulline without ASS1, and were dependent on exogenous arginine (30). Meanwhile, Arginase-1 (ARG1) and arginase-2 (ARG2), which converted arginine to ornithine and urea, were highly expressed in various tumors such as breast cancer and neuroblastoma, as well as cancer-associated cells such as fibroblasts and myeloid-derived suppressor cells (MDSCs). Arginine deficiency in tumor microenvironment impaired T cell responses, and high arginine levels increased T cell survival and anti-tumor effects. Arginine supplementation or targeted ASS1 inhibition might relieve the immunosuppression and reduced T cell dysfunction (31). Another study indicated that baseline arginine levels could predict the survival of cancer patients treated with immune checkpoint inhibitors. In both discovery and validation cohorts, low arginine levels at baseline were significantly associated with the worse benefit rate, progression-free survival, and overall survival (32).

### 3.3 Cysteine and cystine

Cysteine was not an essential amino acid, but it was necessary in synthesis of protein, glutathione, and coenzyme A. Cysteine existed in its oxidized form, cystine, in the oxidative extracellular environment (20). Cystine was transported through the system xc^-^ transporter including SLC3A2 and SLC7A11 subunits, and immediately reduced into cysteine (33). Cysteine was the essential amino acid for T-cell activation because T cells lacked cystathionase, which converted methionine to cysteine. Meanwhile, T cells did not have the xc^−^ transporter and therefore failed to import cystine. T cells depended on macrophages and dendritic cells to export cysteine, which was imported *via* the ASC neutral amino acid transporter, while myeloid-derived suppressor cells could exhaust extracellular cysteine and cystine to block T cell activation (34).

### 3.4 Glutamine

In addition to glucose, glutamine provided both carbon and nitrogen which were necessary for anabolic metabolism, which had long been an attractive therapeutic target for cancer therapy. Blocking glutamine metabolism by glutamine antagonist would not only inhibit tumor cells but also relieve the important immunometabolic checkpoint and restore antitumor immunity. One study indicated glutamine blockade in tumor-bearing mice suppressed glycolytic metabolism in cancer cells, leading to decreased hypoxia and acidosis. Meanwhile, effector T cells responded to glutamine antagonism by markedly increasing oxidative metabolism and adopting highly activated phenotype. Furthermore, tumor-bearing mice treated with concurrent dosing of the glutamine antagonist and anti-PD-1 antibody showed dramatically improved antitumor effects compared with that using anti-PD-1 therapy alone (35). Another study confirmed that inhibition of glutamine in cancer cells synergized with immune checkpoint inhibitor to promote antitumor immunity. Under glutamine-limited conditions, reduced cellular glutathione caused increased PD-L1 expression by impairing SERCA activity. In combination with anti-PD-L1 therapy, glutamine depletion obviously increased the antitumor efficacy of T cells due to simultaneous increases in Fas/CD95 expression (36, 37).

SLC1A5 was the important transporter which mediated glutamine transportation. In addition, many other transporters could mediate glutamine uptake such as SLC6A14, SLC6A19, SLC38A1, SLC38A2, SLC38A4, and SLC38A3 (38, 39). Enhanced glutamine uptake changed composition of immune cell infiltrates in breast cancer, and high expression of glutamine transporters was significantly associated with PD-L1^+^ and PD1^+^, FOXP3^+^, CD68^+^ and CD20^+^ cells, which always indicated poor outcome in cancer patients (40).

### 3.5 Leucine

Leucine was a large neutral amino acid, which had the reputation for its unique function in activating the mTOR signaling pathway (41). Some studies demonstrated that B cells in colorectal cancer (CRC) had an addiction for leucine, which promoted *in vitro* induction of LARS2 and TGF-β1 expression. *In vivo*, the generation of LARS2-expressing B cells was enhanced when applying a leucine-rich diet to mouse models of CRC, which was associated with an increased tumor burden (42–45).

Leucine transportation was mainly mediated by SLC7A5 and SLC3A2 subunits. The two subunits were both upregulated during T cell activation, and SLC7A5 inhibition impaired human T cell activation and function (46).

### 3.6 Methionine

Methionine was an essential sulfur-containing amino acid, which was required for protein synthesis and the S-adenosyl-methionine production. Methionine played an important role in the immune system and dietary supplementation proved to increase mammalian host immunity. By generating S-adenosylmethionine, methionine was a universal methyl donor for DNA, RNA, and protein methyltransferases (47, 48). Some studies found that dietary methionine restriction decreased tumor growth and enhanced antitumor immunity by increasing tumor-infiltrating CD8^+^ T cells in different mouse models. Mechanistically, the S-adenosylmethionine derived from methionine metabolism increased the N^6^-methyladenosine methylation and translation of immune checkpoints such as PD-L1 and VISTA in tumor cells. The tumor methionine metabolism exerted significant impacts on tumor immunity, and two tumor methionine metabolites, S-adenosylmethionine and 5-methylthioadenosine, might promote immune dysfunction in hepatocellular carcinoma. S-adenosylmethionine and 5-methylthioadenosine directly reprogramed the global chromatin accessibility of T cells, and made the T cells exhaustion in tumor microenvironment (49, 50).

### 3.7 Serine

Serine was a non-essential amino acid that could be *de novo* synthesized from glucose. Exogenous serine was necessary for cell proliferation in Ewing sarcoma and liposarcoma. Similarly, serine uptake was required for T cell proliferation, and the

majority of intracellular serine came from extracellular environment (51, 52). Serine stimulated glutathione synthesis and was involved in one-carbon metabolic network that was essential for effector T cells. Glutamate cysteine ligase-deficient Tregs showed increased serine metabolism, mTOR activation, and proliferation but decreased FoxP3 expression. Limitation of cellular serine restored FoxP3 expression of Tregs, which indicated glutathione restricted serine metabolism to preserve Tregs function (53).

### 3.8 Tryptophan

As an essential amino acid, tryptophan was critical for T cell-mediated immunity and considered to be a precursor for 5-hydroxytryptamine and kynurenine. Meanwhile, in isocitrate dehydrogenase (IDH)-mutant gliomas with abnormal tryptophan metabolism, differentiation in infiltrating myeloid cells was blocked, which resulted in an immature phenotype (54). Three enzymes including IDO1, IDO2, and TDO catalyzed tryptophan to formyl-kynurenine. Both IDO2 and TDO were low expressed, and IDO1 was considered to be the predominant form and had been widely studied. High IDO1 expression was associated with poor prognosis in a range of cancers. IDO1 depleted tryptophan and increased kynurenine in the tumor microenvironment, which decreased tumor-infiltrating T cell activity due to GCN2 kinase activity. Meanwhile, kynurenine decreased effector T cell proliferation and supported the Tregs through aryl hydrocarbon receptor. Furthermore, tryptophan-derived microbial metabolites activated aryl hydrocarbon receptor in tumor-associated macrophages to suppress anti-tumor immunity. Therefore, IDO1 inhibition was a target for anti-cancer therapy in order to restore tumor immunity, and several IDO1-inhibitors were currently tested *in vitro* as well as in clinical trials. However, IDO-1 inhibitors might dampen T cell-mediated immunity by limiting tryptophan metabolism, and it was crucial to target IDO-1 inhibitors to cancer cells specifically (55–58).

### 3.9 Other amino acids

Mitochondrial respiration was critical for cell proliferation. In addition to producing ATP, respiration generated biosynthetic precursors, such as aspartate, an essential substrate for nucleotide synthesis. One study showed that in addition to depleting intracellular aspartate, electron transport chain inhibition depleted aspartate-derived asparagine, and impaired mTOR complex I (mTORC1) (59, 60).

Glutamate was a major excitatory neurotransmitter in the mammalian central nervous system. It acted *via* two classes of receptors, ligand gated ion channels and G protein-coupled receptors. GRM4 was expressed at high levels in central nervous system and played an important role in various physiological and pathophysiological processes. GRM4 was a negative regulator of antitumor immunity and targeting of GRM4 might represent a novel strategy to improve cancer immunotherapy. In another study, dysregulation of glutamate transport enhances Tregs function that promoted VEGF blockade resistance in glioblastoma (61, 62).

## 4 Conclusion

Amino acids were the important nutrient for immune cells, which supported protein synthesis to control immune cell function. Different immune cell selectively acquired amino acids, controlled by different antigen, cytokine signaling, and amino acid transporter expression, rather than their indiscriminate uptake. Meanwhile, tumors increased amino acid metabolism to maintain proliferation, even in the nutrient-poor conditions of the tumor microenvironment. Most studies regarding tumor amino acid metabolism focused on the regulatory mechanism, which contributed to tumor growth, epigenetic modification, and tumor immunity. Based on these studies, many novel strategies for cancer therapy have been developed in clinical practice. The strategies included directly targeting amino acids, amino acid transporters and enzymes in amino acid metabolism, and combined with other treatment.

The strategies targeting amino acid transporters provided a novel anti-tumor target. L-type amino acid transporters (LATs) carried neutral amino acids including L-leucine into cells, which stimulated rapamycin complex 1 signaling and protein synthesis. LAT1 or LAT3 knockdown in castration-resistant prostate cancer xenografts decreased tumor growth and spontaneous metastasis *in vivo* (63). Meanwhile, the transporters ASCT2 and LAT1 were overexpressed in aggressive cancers. Genetic disruption of ASCT2 obviously decreased tumor growth in several cancer cell lines (64).

The strategies targeting amino acid enzymes provided another novel anti-tumor target. LY3381916 was the orally available, highly selective inhibitor of IDO1, and one study detected the safety and antitumor activity of LY3381916 monotherapy and in combination with PD-L1 inhibitor in patients with advanced solid tumors (65). Dose-limiting toxicities included increased alanine aminotransferase/aspartate aminotransferase and systemic inflammatory response syndrome. LY3381916 combined with PD-L1 inhibitor presented maximal inhibition of IDO1 activity and increased CD8^+^ T cells infiltration in tumor tissue. However, the clinical activity was limited in this study, and best response was stable disease.

Epacadostat (IDO1 selective inhibitor) and pembrolizumab showed promising antitumor activity in the early phase ECHO-202/KEYNOTE-037 study in advanced melanoma. In the randomised, placebo-controlled, double-blind phase 3 trial, 706 patients were randomly assigned to receive epacadostat plus pembrolizumab or placebo plus pembrolizumab (66). However, no significant differences were found between the treatment groups for progression-free survival or overall survival. The effects of IDO1 inhibition as a strategy to enhance anti-PD-1 therapy activity in cancer remained uncertain.

Despite many setbacks in early clinical trials, targeting amino acid metabolism in cancer cells and immune cells was still a potential strategy in augmenting immune responses, and future research was needed to fill the gaps in using amino acid metabolism for cancer immunotherapy.

## Author contributions

MY wrote the draft of the manuscript. SZ contributed to review, revise and edit the manuscript. All authors contributed to the article and approved the submitted version.
